# Neodymium isotopes in modern human dental enamel: An exploratory dataset for human provenancing

**DOI:** 10.1016/j.dib.2021.107375

**Published:** 2021-09-16

**Authors:** Esther Plomp

**Affiliations:** Faculty of Applied Sciences, Delft University of Technology, Delft, the Netherlands

**Keywords:** Neodymium, Strontium, Isotope, Human, Enamel, Forensic provenancing, the Netherlands

## Abstract

This collection presents data on neodymium isotopes from modern dental elements (third molars) of 47 individuals born and raised in the Netherlands, Grenada, Curaçao, Bonaire, Columbia and Iceland. Neodymium isotope composition was successfully analyzed for 40 individuals (ranging between 0.511820 and 0.512773 ^143^Nd/^144^Nd and -16.0 to 2.6 εNd), with neodymium concentration data available for 23 individuals (ranging between 0.1 and 21.0 ppb). For 37 individuals the dental elements have also been analyzed for strontium isotopes. All analyses were performed on a Thermo Scientific Triton Plus TIMS. Neodymium analyses were performed using 10^13^ Ω resistors, with samples reanalyzed using 10^11^ Ω resistors if enough sample was available. Strontium analyses were performed using 10^11^Ω resistors. A discussion about the applicability of the analysis technique and the results can be found in the article “*Evaluation of neodymium isotope analysis of human dental enamel as a provenance indicator using 10^13^ Ω amplifiers (TIMS)*”. This dataset is available for verification of the provenance capability of neodymium isotope analysis in archaeological and forensic mobility studies. To ensure the interoperability and reusability of the data, the data is available on the IsoArcH (https://isoarch.eu/) data repository.

**Specifications Table**

**Table:** 

Subject	Social Sciences – Archaeology
Specific subject area	Isotope analysis, geochemistry, forensic provenancing, mobility
Type of data	Table Figure Dataset Software
How data were acquired	Data were generated using Thermal Ionization Mass Spectrometry (TIMS). All analyses were performed on a Thermo Scientific Triton Plus TIMS. Neodymium analyses were performed using 10^13^ Ω resistors, with samples reanalyzed using 10^11^ Ω resistors if enough sample was available. Strontium analyses were performed using 10^11^Ω resistors.
Data format	Raw
Parameters for data collection	In order to assess the variability introduced by the laboratory procedures a synthetic tooth standard (TSTD) was used. Standards (CIGO, JNdi-1, NBS987) were also measured to check for accuracy and reproducibility of the measurements. A minimum of 70 scans were collected for each neodymium isotope analysis on 10^13^ Ω amplifiers, and 60 scans for 10^11^ Ω amplifiers.
Description of data collection	Neodymium and strontium isotope analysis was performed on third molars from 47 individuals who were known to have been residentially stable during the period of third molar formation and mineralization (between the age of 8–16 years). Only dental elements unaffected by dental diseases such as caries were selected for analyses.
Data source location	Institution: Faculty of Science, Vrije Universiteit Amsterdam City/Town: See Table 1. Country: the Netherlands, Grenada, Curaçao, Bonaire, Columbia, Iceland Longitude: -72.4985, 6.895 to Latitude: 7.9011, 64.1376
Data accessibility	Repository: IsoArcH [1] Data identification number: 10.48530/isoarch.2021.011 Direct URL: 10.48530/isoarch.2021.011 Software availability: https://doi.org/10.5281/ZENODO.5150520[6] Data is available under the Creative Commons BY-NC-SA 4.0 license.
Related research article	E. Plomp, I.C.C. von Holstein, J.M. Koornneef, R.J. Smeets, J.A. Baart, T. Forouzanfar, G.R. Davies, Evaluation of neodymium isotope analysis of human dental enamel as a provenance indicator using 10^13^ Ω amplifiers (TIMS), Science & Justice (2019). https://doi.org/10.1016/j.scijus.2019.02.001

**Value of the Data**

• Multi-isotopic analyses can provide more specific location estimates than single isotopic analysis. The addition of more isotopic systems, such as neodymium isotope analysis, could provide additional provenance information in the study of human mobility. This neodymium isotope dataset contains information on neodymium concentration and composition of modern human tissues and offers essential comparative material to future neodymium analyses of human tissues.
• The data will be of value in particular to archaeological and forensic studies that investigate geographic mobility. Neodymium isotope analysis is particularly promising for application in coastal regions, as the system is less influenced by the isotopic values of the oceans in comparison to other isotopic systems used for human provenancing.
• This dataset is particularly valuable for reuse as it is time consuming and costly to generate new neodymium isotope data for human tissues. The analysis of neodymium isotopes in human tissues is restricted by the large sample size required (due to the low neodymium concentrations of <21 ppb in human tissues) and the need for the latest analytical techniques (10^13^ Ω amplifiers).
• Once neodymium isotope analysis becomes more generally applicable due to technical developments, this data may eventually be used to generate maps displaying the spatial distribution of isotopic values, known as isoscapes. The strontium isotope data can already be used for these purposes.

## 1 Data Description

**Fig 1:**
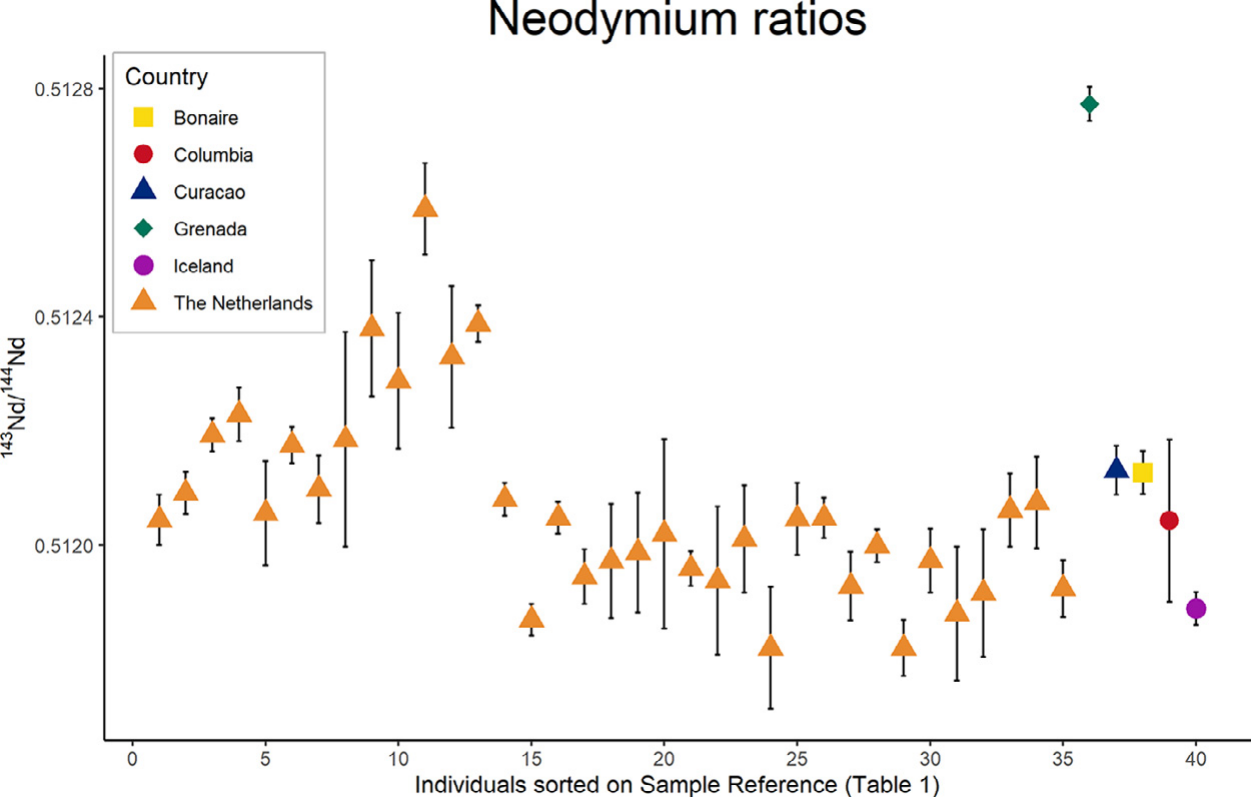
Nd isotope composition of modern third molars of 40 individuals. Underlying code of the figure is available [6] and made use of scripts by Stantis [7]**.**

This collection presents the neodymium (Nd) and strontium (Sr) isotope results from modern human dental elements (third molars) of 47 individuals born and raised in the Netherlands, Grenada, Curaçao, Bonaire, Columbia and Iceland [2] (Table 1). Neodymium isotope composition was successfully analyzed for 40 individuals (ranging between ^143^Nd/^144^Nd = 0.511820 to 0.512773) (Fig. 1). The ^143^Nd/^144^Nd results were converted to εNd values to make the small differences in the ^143^Nd/^144^Nd values more apparent. This conversion also facilitates comparison with other datasets that shared εNd values (see for example [3]). εNd is calculated as following:

$$\varepsilon_{\text{Nd}}\left(\frac{{\left({}^{143}\text{Nd}/{}^{144}\text{Nd}\right)}_{\text{sample}}}{{\left({}^{143}\text{Nd}/{}^{144}\text{Nd}\right)}_{\text{CHUR}}}-1\right)\times 10^{4}$$

**Table 1:** Nd concentration and Nd and Sr isotope composition of modern third molars, modified from [2]. Detailed spatial information is available in [5].

Sample Reference	Individual Reference	Site Reference	Country	^87^Sr/^86^Sr	SD	^143^Nd/^144^Nd	SD	εNd	Nd (ppb)	Literature Reference
1	1-A22	Amsterdam	the Netherlands			0.512044	0.000044	-11.6		24
2	2-A20	Warmenhuizen	the Netherlands	0.709267	0.000007	0.512091	0.000037	-10.7	1.3	11, 24
3	3-A24	Amsterdam	the Netherlands	0.709378	0.000007	0.512193	0.000029	-8.7	2.3	11, 24
4	4-AH	Purmerend	the Netherlands			0.512229	0.000047	-8.0	19.8	24
5	5-A25	Amsterdam	the Netherlands			0.512056	0.000091	-11.4	0.7	24
6	6-A10	Amsterdam	the Netherlands	0.709315	0.000006	0.512175	0.000032	-9.0	3.1	11, 24
7	7-A13	Amsterdam	the Netherlands	0.709409	0.000009	0.512098	0.000059	-10.5	0.4	11, 24
8	8-A27	Alkmaar	the Netherlands	0.709367	0.000007	0.512185	0.000188	-8.8		11, 24
9	9-A28	Amsterdam	the Netherlands			0.512380	0.000119	-5.0		24
10	10-A18	Amsterdam	the Netherlands	0.709584	0.000006	0.512288	0.000119	-6.8		11, 24
11	11-A15	Amsterdam	the Netherlands	0.709441	0.000004	0.512589	0.000080	-1.0		11, 24
12	12-A9	Amsterdam	the Netherlands	0.709231	0.000004	0.512330	0.000124	-6.0	1.1	11, 24
13	28-R14a	Dordrecht	the Netherlands	0.709153	0.000006	0.512388	0.000032	-4.9	21.0	24
14	29-R11	Rotterdam	the Netherlands	0.709375	0.000011	0.512080	0.000029	-10.9		24
15	30-R13	Rotterdam	the Netherlands	0.709409	0.000009	0.511869	0.000028	-15.0		24
16	31-R2	Rotterdam	the Netherlands			0.512048	0.000028	-11.5		24
17	32-R3	Rotterdam	the Netherlands			0.511945	0.000048	-13.5		24
18	33-R9	Rotterdam	the Netherlands			0.511972	0.000100	-13.0		24
19	34-R5	Rotterdam	the Netherlands	0.709061	0.000008	0.511987	0.000105	-12.7		24
20	35-R9	Rotterdam	the Netherlands	0.709821	0.000009	0.512020	0.000166	-12.1		24
21	36-F1	Lippenhuizen	the Netherlands	0.709432	0.000009	0.511959	0.000030	-13.2		2
22	37-F3	Holwerd	the Netherlands	0.709619	0.000009	0.511938	0.000130	-13.7		2
23	38-F4	Leeuwarden	the Netherlands	0.708934	0.000010	0.512011	0.000094	-12.2		2
24	39-F8	Leeuwarden	the Netherlands	0.709469	0.000007	0.511820	0.000107	-16.0		2
25	40-F11	Leeuwarden	the Netherlands	0.709230	0.000009	0.512046	0.000063	-11.5		2
26	41-F12	Oldeboorn	the Netherlands	0.709122	0.000009	0.512048	0.000035	-11.5		2
27	42-F13	Leeuwarden	the Netherlands	0.709337	0.000009	0.511928	0.000060	-13.8		2
28	43-R6	Maastricht	the Netherlands	0.708942	0.000007	0.511999	0.000029	-12.5		2
29	44-M4	Maastricht	the Netherlands	0.709596	0.000007	0.511820	0.000049	-16.0		2
30	45-M5	Maastricht	the Netherlands	0.709644	0.000010	0.511973	0.000056	-13.0		2
31	47-M14	Maastricht	the Netherlands	0.709546	0.000010	0.511880	0.000117	-14.8	0.1	2
32	48-ZH1	Heerlen	the Netherlands	0.709424	0.000008	0.511916	0.000112	-14.1	0.1	2
33	49-ZH3	Heerlen	the Netherlands	0.709319	0.000009	0.512061	0.000064	-11.3	0.4	2
34	50-ZH4	Heerlen	the Netherlands	0.709169	0.000009	0.512075	0.000080	-11.0	0.9	2
35	51-ZH9	Vaals	the Netherlands	0.709862	0.000008	0.511924	0.000050	-13.9	0.5	2
36	W1-Gr	St. George's	Grenada	0.707841	0.000009	0.512773	0.000030	2.6	7.9	2
37	W2-R8	Willemstad	Curaçao	0.709375	0.000010	0.512131	0.000043	-9.9		2
38	W3-B4	Kralendijk	Bonaire	0.709256	0.000009	0.512127	0.000038	-10.0	0.6	2
39	W4-B16	Cúcuta	Columbia	0.711749	0.000009	0.512043	0.000142	-11.6	0.2	2
40	W5-I	Reykjavik	Iceland	0.708740	0.000009	0.511889	0.000029	-14.6		2
41	46-M10	Maastricht	the Netherlands						0.1	2, 24
42	52-7	Utrecht	the Netherlands	0.709382	0.000006				0.5	2, 11, 24
43	53-3	Maarssen	the Netherlands	0.708951	0.000006				1.8	2, 11, 24
44	54-S3a	Kortgene	the Netherlands						0.8	2, 24
45	55-S2b	Kortgene	the Netherlands						0.8	2, 24
46	56-1	Den Bosch	the Netherlands	0.709611	0.000009				1.2	2, 11, 24
47	57-16	Enschede	the Netherlands	0.709674	0.000008				0.5	2, 11, 24

**Fig. 2:**
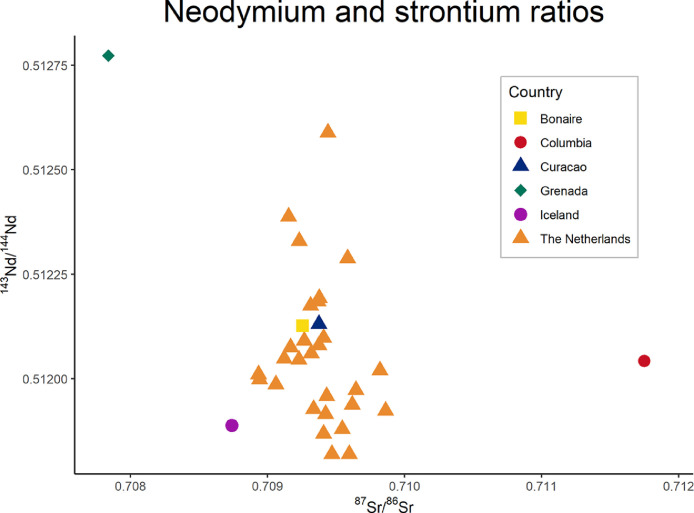
Nd and Sr isotope compositions of modern third molars of the 33 individuals that were analyzed for both Nd and Sr. Standard deviations are available in Fig. 1 and Table 1. Underlying code of the figure is available [6] and made use of scripts by Stantis [7].

Where CHUR is the Chondritic Uniform Reservoir (CHUR, ^143^Nd/^144^Nd = 0.512638) [4]. εNd values in this dataset ranged from -16.0 to 2.6. Neodymium elemental concentration ranged between 0.1 and 21.0 ppb (*n* = 23). Strontium isotope results are available for 37 individuals (ranging between ^87^Sr/^86^Sr = 0.707841 to 0.711749). Combined Sr-Nd isotope results were obtained for 37 individuals (Fig. 2). The full dataset described in Table 1 is available on IsoArcH [1] in .xlsx format and includes more detailed geographical information of the samples (latitude, longitude, altitude and distance from sea) as well as a .ris file containing the relevant research articles [5].

To evaluate whether Nd isotope analysis can be used as a human provenancing technique this dataset should be compared to local isotopic ranges. Previous ^143^Nd/^144^Nd estimates (based on sediment data) for the Netherlands range from ^143^Nd/^144^Nd = 0.51198 to 0.51217 (*n* = 18) [8,9]. The Dutch ^87^Sr/^86^Sr range, based on primarily human enamel data, lies between 0.709 and 0.710 [10,11]. The Maraicaibo Basin area (Cúcuta) isotopic data is based on models that estimate the local Sr range >0.710 [12] and ^143^Nd/^144^Nd ratios around ∼0.5120 [13]. The estimated geological isotopic ranges for Bonaire and Curaçao are ^87^Sr/^86^Sr = 0.703–0.709 and ^143^Nd/^144^Nd = 0.5120-0.5130 [14], [15], [16]. Grenada geological isotopic ratios are expected to be in the range of ^143^Nd/^144^Nd = 0.5123–0.5126 and ^87^Sr/^86^Sr = 0.7038–0.7064 [17]. Bonaire, Curaçao and Grenada are influenced by dust flux from North Africa (^143^Nd/^144^Nd = 0.5116- 0.5126, ^87^Sr/^86^Sr = 0.715–0.718) [18], [19], [20], [21] and sea-spray influence (^87^Sr/^86^Sr = 0.7092). Based on geological research the Reykjavik region is also influenced by sea-spray and the ^143^Nd/^144^Nd and ^87^Sr/^86^Sr ratios of its geology are expected to range between 0.5130 to 0.5131 and 0.7031 to 0.7032 [22,23]. The isotope results from the individuals described in Table 1 are either (1) consistent with the local geology in both Sr and Nd isotopes (Colombia), (2) consistent with local Sr geology but not always with the expected Nd ratios (the Netherlands), (3) consistent with local geology in Nd isotopes but with elevated ^87^Sr/^86^Sr ratios (Grenada, Curaçao and Bonaire) or (4) incompatible with the local geology in both Sr and Nd isotope ratios (Iceland). For a more detailed comparison and interpretation of these background and human isotopic ranges, please see Plomp et al. [2].

## 2 Experimental Design, Materials and Methods

### 2.1 Sample preparation

The enamel was sampled, chemically processed and analyzed at the Faculty of Science, Vrije Universiteit Amsterdam [2,11,24]. Teeth were collected in cleaned 50 mL plastic centrifuge tubes (rinsed >3 times with Milli-Q and 1 time with ethanol (Purity Grade: absolut, CHROMASOLV®, for high-performance liquid Chromatography)). Prior to sampling, the teeth were leached overnight in 30% H_2_0_2_ (Sigma-Aldrich Company Ltd), rinsed in ultrapure water (Milli-Q) and air dried on a hotplate at 50 °C. Afterwards, the enamel was sampled using a dental micro-drill fitted with a cleaned diamond tipped rotary burr and blade (Minilor Perceuse). Care was taken to mechanically remove any dentine for the enamel closer to the enamel-dentine junction to ensure that only enamel was sampled. The burr and blade were cleaned between sampling with 3 N HNO_3_ in an ultrasonic bath for 3 minutes to remove any residual particles and then rinsed with Milli-Q and ethanol, and afterwards dried before sampling the next molar. Enamel was collected on clean aluminium foil (new foil was used for each sample) before transferring the sampled enamel to clean glass vials. If two third molars were available from a single donor, the enamel from both teeth was combined to increase the available sample size. Sample weight for Nd composition samples ranged from 222 to 1464 mg (average = 733 mg, *n* = 20) [2]. Enamel samples were dissolved using perfluoroalkoxy (PFA) laboratory equipment, which was cleaned according to standard procedures [24,25]. All PFA vials were first sub-boiled in bulk in pro-analysis quality 7 N HNO_3_ and 6 N HCl for two hours each. The inside of the vials were cleaned through two leaching steps at 125 °C with (1) double distilled 6.5 N HCl (>5 days) and (2) 7 N HNO_3_/12 N HF (>2 days).

For 25 individuals an aliquot (1–2%) was taken for Sr analysis (Zuid-Holland, Limburg, Friesland, Iceland, Grenada, Curaçao, Bonaire and Columbia [2]). For the remaining 12 individuals the Sr isotope analysis took place on a separate sampled section of the enamel, following the protocol by Font et al. [11].

### 2.2 Sample processing

In order to determine the range of Nd concentrations in human teeth, isotope dilution (using a ^150^Nd enriched spike (^150^Nd/^144^Nd = 142.93)) was performed on a subset of the samples before dissolution [2]. Isotope dilution allows for measurement of both neodymium concentration and composition of a sample. Sampled enamel was dissolved in 6.5 N HCl (3–6 mL, depending on sample size), dried and nitrated before being re-dissolved in a mixture of 6.5 N HCl and 14.0 N HNO_3_ (3–6 mL, depending on sample size). Afterwards the samples were dried, nitrated and re-dissolved in 10 mL 2.0 N HNO_3_ for column extraction [26]. An aliquot of 100–200 µL (depending on sample size) was taken from the samples for Sr analysis, which was separated using pipette tips (with 30 µm pore size frit material) and 100 µL Sr-spec resin [25]. Neodymium was extracted using TRU-resin columns (Pasteur pipettes, 35 µm polyethelene frit) with resin volumes ranging from 0.75 mL (samples<550 mg) to 1.3 mL (samples>550 mg). Before loading the samples on the columns they were ultrasonicated (30 minutes) and centrifuged (4000 rpm, 4 minutes). TRU-resin columns were prepared according to the following steps:

**Cleaning of TRU-resin columns:**

6 mL 2 N HF

6 mL Milli-Q

6 mL 2 N HNO_3_

6 mL Milli-Q

**Precondition:**

6 mL 2 N HNO_3_

**Prefraction (25 Column Volume - CV):**

19 or 33 mL 2 N HNO_3_ (depending on 0.75 or 1.3 mL TRU-resin)

Sample load: 10 mL 2 HNO_3_

Wash: 9 or 23 mL 2 N HNO_3_

**REE extraction (10 CV):**

8 or 14 mL Milli-Q

After the first extraction in 8 mL (samples <550 mg) or 14 mL (samples <550 mg) Milli-Q, the REE fraction was collected and reloaded onto the column for a second purification, as enamel is calcium rich and may overload the columns which could result in incomplete removal of calcium in the first purified fraction. After the second collection the REE fraction was dried overnight on a hotplate at 120 °C. After Light Rare Earth Element (LREE) extraction, Nd was separated from the other LREE using Ln-resin (Eichrom Technologies) following standard procedure [2,26], described in detail below:

**Cleaning of Ln-resin columns day 1:**

4 mL 6 N HNO_3_

4 mL 2 N HF

4 mL Milli-Q

4 mL 6-7 N HCl

1 mL 0.165 N HCl

(store columns overnight in 0.165 N HCl in centrifuge tubes (10 mL, cleaned with 6–7 N HCl for > 7 days))

**Cleaning of Ln-resin columns day 2:**

2 mL 6-7 N HCl

2 mL Milli-Q

**Precondition:**

2 mL 0.165 N HCl

**Prefraction:**

9–11 mL 0.165 N HCl (depending on how long the Ln-resin is in use)

Sample load: 1–2 mL 0.165 N HCl

Sample wash: 7–10 mL 0.165 N HCl

**Nd extraction:**

4 mL 0.3 N HCl

After Nd extraction in 4 mL 0.3 N HCl the samples were dried down and nitrated with 10 drops of concentrated HNO_3_, fluxed for 2 hours at 120 °C with the PFA caps closed, and then dried down at 110 °C for TIMS analysis.

In order to assess the variability introduced by the laboratory procedures a synthetic tooth standard was used [2]. As Nd concentration in human teeth is low (<21 ppb) it was not viable to create a standard by combining a large amount of human teeth. The synthetic tooth standard (TSTD) was created using 400 g CaHPO_4_ (Alfa Aesar) dissolved in 4 L 3 N HNO_3_, doped with 100 ppm Sr (Alfa Duchefa, 1000 ppm Sr, ICP standard code: 970504), 1 ppm Pb (CPI International, 1000 ppm Pb, ICP standard code: P/N 4400–1000281), and 4 ppb Nd (Alfa Aesar, ICP standard code: 9301120). Aliquots of this standard were processed on 0.75 and 1.3 mL TRU-resin columns (10 mL, 4 ng Nd, 1000 mg CaHPO_4_) and Sr columns (0.05 mL, 500 ng Sr, 5 mg CaHPO_4_) [26].

The Sr aliquots and standards analyzed by Plomp et al. [2] were processed using reusable Sr pipette columns (frit material 30 µm, 100 µL Sr resin) [25]. Sr columns are first rinsed 3 times with Milli-Q before adding the Sr-resin. Before being loaded onto the columns the samples are ultrasonicated and centrifuged in the same manner as described for the Nd fraction.

**Cleaning of Sr-resin low blank columns:**

1 CV 3 N HNO_3_

1 CV 1-2 N HF

1 CV Milli-Q

1 CV 3 N HNO_3_

1 CV Milli-Q

1 CV 3 N HNO_3_

1 CV Milli-Q

**Precondition:**

0.5 CV 3 N HNO_3_

**Prefraction (30 CV / 3 mL):**

Sample load: 0.5 mL 3 N HNO_3_ / Tooth Standard load: 0.05 mL (500 ng Sr, 5 mg CaHPO)

Wash: 2.5 mL 3 N HNO_3_ / Wash: 2.95 mL 3 N HNO_3._

**Sr extraction (10 CV / 1 mL):**

1 mL Milli-Q (collect in clean beakers)

After Sr extraction the columns have to be cleaned before storing them for future use (removing the resin and rinsing with Milli-Q).

The Sr samples analyzed by Font et al. [11] followed a similar low blank Sr column protocol. For these samples the enamel was first dissolved in 2 mL of 14 N HNO_3_ for 12 hours on a hotplate at 110 °C. After drying down the samples were re-dissolved in 1 mL 3 N HNO_3_, which was processed on disposable Sr columns (pipette tips with frit material 30 µm and 100 µL Sr spec resin).

### 2.3 Sample analysis

Neodymium and Sr analyses were performed on a Thermo Scientific Triton Plus Thermal Ionization Mass Spectrometry (TIMS). Standards and samples were loaded on outgassed Re filaments in 1–2 µL 10% HNO_3_ with 1 µL H_3_PO_4_ for Nd and 50% of the Sr fraction in 1 µL 10% HNO_3_, with 1.5 µL TaCl_5_ for Sr. Neodymium analyses were performed using 10^13^ Ω resistors fitted to the amplifier system and 10^11^ Ω resistors if enough sample was available [2]. The 10^13^ Ω resistors measurements result in a 100 fold higher output voltage compared to default 10^11^ Ω resistors (while the signal to noise ratio is improved by a factor of 10). This allows for more precise measurements of small data using the 10^13^ Ω resistors.

Strontium analyses were performed using 10^11^Ω resistors. Mass-fractionation corrections were performed to ^146^Nd/^144^Nd = 0.7219 and ^86^Sr/^88^Sr = 0.1194. Standards were measured to check for accuracy and reproducibility (Table 2). Blank corrections were not necessary as total procedural blanks yielded negligible amount of neodymium (1.1 ± 1.7 pg, *n* = 56) and strontium (24.7 pg ± 38.9, *n*= 26). Blank data for the samples by Font et al. [11] was not reported but is assumed to be similarly low as the lead blanks reported in study (≤50 pg Pb, *n* = 27).

**Table 2:** Results of standards measured during the data collection period. JNdi-1 and NBS987 are international standards. CIGO and TSTD are inhouse standards.

Standard	Quantity	Amplifier	^143^Nd/^144^Nd	2 SD	*n*	^87^Sr/^86^Sr	2 SD	n
CIGO	0.1 ng	10^13^	0.511344	70	40			
	250 ng	10^11^	0.511328	9	50			
JNdi-1	200 ng	10^11^	0.512096	61	22			
TSTD	0.5–4.0 ng	10^13^	0.512134	72	81			
	3–4 ng Nd; 100–200 ng Sr	10^11^	0.512125	61	49	0.707854	19	97
NBS987	100-200 ng					0.710247	17	51

## Ethics Statement

Informed consent was given for each sample donation. Survey data was collected anonymously where possible, with location information limited to the place of birth and age indication rather than exact birth dates. These personal data were processed in accordance to the General Data Protection Regulation (GDPR)/Algemene verordening gegevensbescherming (AVG). The Medical Ethics Review Committee of the Amsterdam UMC (location VUmc) approved the sampling request (IDIS 2010/265).

## CRediT Author Statement

**Esther Plomp:** Methodology, Data curation, Writing – review & editing.

## Declaration of Competing Interest

The author declares that they have no known competing financial interests or personal relationships which have influenced the work reported in this article. The author is the Open Research Ambassador of IsoArcH.

## References

[1] Salesse K., Fernandes R., de Rochefort X., Brůžek J., Castex D., Dufour É. (2018). IsoArcH.eu: an open-access and collaborative isotope database for bioarchaeological samples from the Graeco-Roman world and its margins. J. Archaeol. Sci. Rep..

[2] Plomp E., von Holstein I.C.C., Koornneef J.M., Smeets R.J., Baart J.A., Forouzanfar T., Davies G.R. (2019). Evaluation of neodymium isotope analysis of human dental enamel as a provenance indicator using 1013 Ω amplifiers (TIMS). Sci. Justice.

[3] Brems D., Ganio M., Latruwe K., Balcaen L., Carremans M., Gimeno D., Silvestri A., Vanhaecke F., Muchez P., Degryse P. (2013). Isotopes on the beach, part 2: neodymium isotopic analysis for the provenancing of Roman glass-making. Archaeometry.

[4] Jacobsen S.B., Wasserburg G.J. (1980). Sm-Nd isotopic evolution of chondrites. Earth Planet. Sci. Lett..

[5] Plomp E. (2021). Neodymium isotopes in modern human dental enamel: an exploratory dataset. IsoArcH.

[6] E. Plomp, J.C. Peterson, [software] EstherPlomp/figures-Nd-data, Zenodo, 2021. doi:10.5281/ZENODO.5150520.

[7] C. Stantis, [software] stantis/IsoDataVis: first (Official) release, Zenodo, 2021. doi:10.5281/ZENODO.4743734.

[8] Bayon G., Toucanne S., Skonieczny C., André L., Bermell S., Cheron S., Dennielou B., Etoubleau J., Freslon N., Gauchery T., Germain Y., Jorry S.J., Ménot G., Monin L., Ponzevera E., Rouget M.-L., Tachikawa K., Barrat J.A. (2015). Rare earth elements and neodymium isotopes in world river sediments revisited. Geochim. Cosmochim. Acta.

[9] Kuhlmann G., de Boer P.L., Pedersen R.B., Wong T.E. (2004). Provenance of Pliocene sediments and paleoenvironmental changes in the southern North Sea region using Samarium–neodymium (Sm/Nd) provenance ages and clay mineralogy. Sediment. Geol..

[10] Kootker L.M., Plomp E., Ammer S.T.M., Hoogland V., Davies G.R. (2020). Spatial patterns in 87Sr/86Sr ratios in modern human dental enamel and tap water from the Netherlands: implications for forensic provenancing. Sci. Total Environ..

[11] Font L., van der Peijl G., van Leuwen C., van Wetten I., Davies G.R. (2015). Identification of the geographical place of origin of an unidentified individual by multi-isotope analysis. Sci. Justice.

[12] Bataille C.P., Laffoon J., Bowen G.J. (2012). Mapping multiple source effects on the strontium isotopic signatures of ecosystems from the circum-Caribbean region. Ecosphere.

[13] Restrepo-Pace P.A., Ruiz J., Gehrels G., Cosca M. (1997). Geochronology and Nd isotopic data of Grenville-age rocks in the Colombian Andes: new constraints for Late Proterozoic-early Paleozoic paleocontinental reconstructions of the Americas. Earth Planet. Sci. Lett..

[14] Thompson P.M.E., Kempton P.D., White R.V., Saunders A.D., Kerr A.C., Tarney J., Pringle M.S. (2004). Elemental, Hf–Nd isotopic and geochronological constraints on an island arc sequence associated with the Cretaceous Caribbean plateau: Bonaire, Dutch Antilles. Lithos.

[15] Laffoon J.E., Sonnemann T.F., Shafie T., Hofman C.L., Brandes U., Davies G.R. (2017). Investigating human geographic origins using dual-isotope (87Sr/86Sr, δ18O) assignment approaches. PLoS ONE.

[16] Tachikawa K., Arsouze T., Bayon G., Bory A., Colin C., Dutay J.-C., Frank N., Giraud X., Gourlan A.T., Jeandel C., Lacan F., Meynadier L., Montagna P., Piotrowski A.M., Plancherel Y., Pucéat E., Roy-Barman M., Waelbroeck C. (2017). The large-scale evolution of neodymium isotopic composition in the global modern and Holocene ocean revealed from seawater and archive data. Chem. Geol..

[17] White W., Copeland P., Gravatt D.R., Devine J.D. (2017). Geochemistry and geochronology of Grenada and Union islands, Lesser Antilles: the case for mixing between two magma series generated from distinct sources. Geosphere.

[18] Aarons S.M., Aciego S.M., Gleason J.D. (2013). Variable HfSrNd radiogenic isotopic compositions in a Saharan dust storm over the Atlantic: implications for dust flux to oceans, ice sheets and the terrestrial biosphere. Chem. Geol..

[19] Zhao W., Balsam W., Williams E., Long X., Ji J. (2018). Sr–Nd–Hf isotopic fingerprinting of transatlantic dust derived from North Africa. Earth Planet. Sci. Lett..

[20] Chapela Lara M., Buss H.L., Pett-Ridge J.C. (2018). The effects of lithology on trace element and REE behavior during tropical weathering. Chem. Geol..

[21] Pourmand A., Prospero J.M., Sharifi A. (2014). Geochemical fingerprinting of trans-Atlantic African dust based on radiogenic Sr-Nd-Hf isotopes and rare earth element anomalies. Geology.

[22] Peate D.W., Baker J.A., Jakobsson S.P., Waight T.E., Kent A.J.R., Grassineau N.V., Skovgaard A.C. (2009). Historic magmatism on the Reykjanes Peninsula, Iceland: a snap-shot of melt generation at a ridge segment. Contrib. Miner. Pet..

[23] Koornneef J.M., Stracke A., Bourdon B., Meier M.A., Jochum K.P., Stoll B., Grönvold K. (2012). Melting of a two-component source beneath Iceland. J. Pet..

[24] Plomp E., von Holstein I.C.C., Koornneef J.M., Smeets R.J., Font L., Baart J.A., Forouzanfar T., Davies G.R. (2017). TIMS analysis of neodymium isotopes in human tooth enamel using 10^13^ Ω amplifiers. J. Anal. At. Spectrom..

[25] E. Plomp, R. Smeets, G. Davies, [protocol] Chromatographic separation of strontium isotopes in human dental enamel for thermal ionization mass spectrometry (TIMS) analysis v1, protocols.io, 2020. doi:10.17504/protocols.io.37dgri6.

[26] E. Plomp, R. Smeets, J. Koornneef, G. Davies, [protocol] Chromatographic separation of neodymium isotopes in human dental enamel for thermal ionization mass spectrometry (TIMS) analysis v1, protocols.io, 2019. doi:10.17504/protocols.io.xzmfp46.

